# Evaluation of Liver Fibrosis Using Texture Analysis on Combined-Contrast-Enhanced Magnetic Resonance Images at 3.0T

**DOI:** 10.1155/2015/387653

**Published:** 2015-09-01

**Authors:** Takeshi Yokoo, Tanya Wolfson, Keiko Iwaisako, Michael R. Peterson, Haresh Mani, Zachary Goodman, Christopher Changchien, Michael S. Middleton, Anthony C. Gamst, Sameer M. Mazhar, Yuko Kono, Samuel B. Ho, Claude B. Sirlin

**Affiliations:** ^1^Departments of Radiology, University of California, San Diego, CA 92103, USA; ^2^Department of Radiology and Advanced Imaging Research Center, University of Texas Southwestern Medical Center, 2201 Inwood Road, NE2.210B, Dallas, TX 75390-9085, USA; ^3^Computational and Applied Statistics Laboratory, San Diego Supercomputer Center, University of California, San Diego, CA 92093, USA; ^4^Departments of Medicine, University of California, San Diego, CA 92103, USA; ^5^Department of Target Therapy Oncology, Kyoto University Graduate School of Medicine, Kyoto, Japan; ^6^Departments of Pathology, University of California, San Diego, CA 92103, USA; ^7^Department of Pathology, Penn State Hershey Medical Center, Hershey, PA 17033, USA; ^8^Center for Liver Diseases, Inova Fairfax Hospital, Falls Church, VA 22042, USA; ^9^VA San Diego Healthcare System, San Diego, CA 92161, USA

## Abstract

*Purpose*. To noninvasively assess liver fibrosis using combined-contrast-enhanced (CCE) magnetic resonance imaging (MRI) and texture analysis. *Materials and Methods*. In this IRB-approved, HIPAA-compliant prospective study, 46 adults with newly diagnosed HCV infection and recent liver biopsy underwent CCE liver MRI following intravenous administration of superparamagnetic iron oxides (ferumoxides) and gadolinium DTPA (gadopentetate dimeglumine). The image texture of the liver was quantified in regions-of-interest by calculating 165 texture features. Liver biopsy specimens were stained with Masson trichrome and assessed qualitatively (METAVIR fibrosis score) and quantitatively (% collagen stained area). Using *L*_1_ regularization path algorithm, two texture-based multivariate linear models were constructed, one for quantitative and the other for quantitative histology prediction. The prediction performance of each model was assessed using receiver operating characteristics (ROC) and correlation analyses. *Results*. The texture-based predicted fibrosis score significantly correlated with qualitative (*r* = 0.698, *P* < 0.001) and quantitative (*r* = 0.757, *P* < 0.001) histology. The prediction model for qualitative histology had 0.814–0.976 areas under the curve (AUC), 0.659–1.000 sensitivity, 0.778–0.930 specificity, and 0.674–0.935 accuracy, depending on the binary classification threshold. The prediction model for quantitative histology had 0.742–0.950 AUC, 0.688–1.000 sensitivity, 0.679–0.857 specificity, and 0.696–0.848 accuracy, depending on the binary classification threshold. *Conclusion*. CCE MRI and texture analysis may permit noninvasive assessment of liver fibrosis.

## 1. Introduction

The ongoing epidemic of Chronic Liver Disease (CLD) is a major contributor to liver-related mortality and morbidity in the United States. More than 20,000 Americans die from CLD complications each year [1]. The most common etiologies for CLD are chronic hepatitis C virus (HCV) infection and alcoholic hepatitis [2, 3]. Over 4 million Americans are HCV carriers, but many are asymptomatic and unaware of their infection [4].

The common pathway in the natural history of CLD, including chronic HCV infection, is progressive liver fibrosis and ultimately cirrhosis [5]. Fibrosis indicates cumulative liver damage, contributes to the development of portal hypertension and hepatic dysfunction, and predicts poor clinical outcome [6, 7]. Most liver-related mortality and morbidity occur in the cirrhotic population [8]. Assessment of liver fibrosis is therefore critical in the management of patients with CLD.

The current gold standard for fibrosis evaluation is liver biopsy. The severity of fibrosis due to HCV infection is often classified using an ordinal scale such as the Metavir system [9]. However, biopsy is invasive and thus problematic for frequent monitoring. Moreover, its interpretation is subjective, leading to inter- and intraobserver variability [10–12]. For these reasons, noninvasive and objective techniques are under investigation, including fibrosis-specific serum markers [13, 14], ultrasound elastography [15, 16], magnetic resonance (MR) elastography [17–19], diffusion weighted MR imaging [20–22], and single-contrast-enhanced MR imaging [23–25].

**Figure 1:**
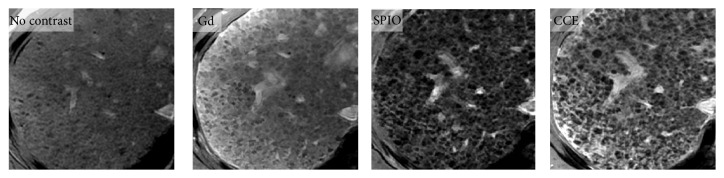
MR images of liver in 60-year old man with HCV-related cirrhosis. Noncontrast, Gd-only, SPIO-only, and CCE 2D breath-hold T1-weighted gradient-echo images of cirrhotic liver due to HCV. Abnormal reticular pattern of the liver parenchyma is better visualized on single-contrast-enhanced (Gd or SPIO) images than on unenhanced image and better visualized on CCE images than on single-contrast-enhanced images. Gd: gadolinium; SPIO: superparamagnetic iron oxide, and CCE: combined contrast enhanced.

**Figure 2:**
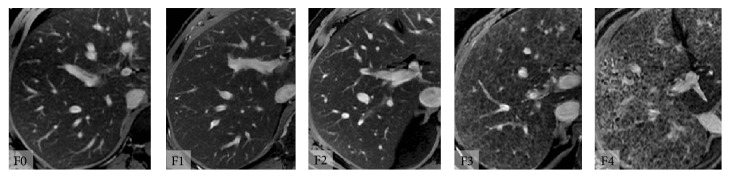
Combined contrast enhanced (CCE) MR images at various stages of fibrosis. CCE MR images in adults with chronic HCV infection and histologically determined Metavir fibrosis stages F0, F1, F2, F3, and F4. Subjectively, the reticular texture of the liver parenchyma becomes progressively more pronounced with increasing Metavir fibrosis stage.

Another promising MR imaging-based technique is combined-contrast-enhanced (CCE) MR imaging [26]. This technique exploits the complementary effects of positive contrast enhancement by gadolinium-chelates (Gd) and negative enhancement by superparamagnetic iron oxide (SPIO) agents. Compared to noncontrast, Gd-enhanced, or SPIO-enhanced images, CCE images better depict the reticular signal abnormalities associated with fibrosis as shown in Figure 1 [27]. The conspicuity of this pattern appears to parallel histologic fibrosis severity (Figure 2) suggesting that liver fibrosis can be assessed by the severity of the “texture” abnormality.

The potential role of texture analysis in liver fibrosis assessment was previously explored in retrospective studies using qualitative [26] and quantitative [26, 28–30] texture analysis. The purpose of this prospective study was to provide proof-of-concept that quantitative texture analysis using CCE MR imaging may permit noninvasively assess liver fibrosis in adults with HCV infection using CCE MR imaging.

## 2. Method and Materials

### 2.1. Study Design and Subjects

This prospective, cross-sectional, observational clinical study was approved by an institutional review board and is HIPAA-compliant. Potential eligible subjects were referred for research MR imaging examination from hepatology clinics at our institution. Written informed consents were obtained. Selection criteria are listed in Table 1. Patient recruitment was stratified according to the fibrosis severity at liver biopsy and continued until at least five subjects in each fibrosis severity category (per clinical biopsy reports) were enrolled.

### 2.2. Liver Biopsy

Subjects had a percutaneous 16-gauge needle-core biopsy of the right hepatic lobe for clinical care by the referring hepatologists. Specimens were processed in the pathology department per routine protocol, including Masson-trichrome staining. Clinical biopsy reports were generated by staff pathologists. Each clinical report included assessment of fibrosis severity (none, mild, moderate, severe, and cirrhosis); the clinically reported fibrosis severity was used for the block recruitment but not analyzed.

### 2.3. Qualitative and Quantitative Scoring of Histology

The trichrome-stained slides were further evaluated for research purposes. The entire slides were digitized using an APERIO ScanScope scanner (Aperio Technologies, Inc., Vista, CA). The digitized images were viewed using the Aperio ImageScope software and the fibrosis severity was scored qualitatively by histomorphology and quantitatively by digital image analysis.

Qualitative scoring was performed independently by three pathologists with expertise in liver pathology (MRP, HM, and ZG). Without knowledge of clinical, MR imaging, or quantitative histology findings, each reader reviewed the digitized histology images, subjectively assessed the adequacy of each specimen, and assigned to each specimen a Metavir fibrosis score, F0–F4. The readers were blinded to each other's scores. Other histology features (e.g., necro-inflammation, steatosis, iron) were not recorded. To assess adequacy of specimen, one pathologist (MRP) counted the number of portal triads within each noncirrhotic specimen; portal triads were not counted in cirrhotic specimens due to architectural distortion. The total length of each specimen was recorded.

Quantitative scoring was performed by a hepatology research scientist (KI) using ImageScope software analysis tools, without knowledge of the clinical, MR imaging, or qualitative histology findings. Staining variability was corrected by digitally adjusting color saturation. Total specimen area was manually segmented, and the blue-stained pixels (representing collagen) were segmented using manual intensity thresholding. Percent (%) collagen was calculated as the ratio of blue-stained to total specimen pixels.

### 2.4. MR Imaging

Subjects received SPIO (ferumoxides, Feridex, Bayer HealthCare Pharmaceuticals, Wayne, NJ) continuous intravenous infusion (0.5 mL/kg) diluted in 100 mL of 5% dextrose solution, passed through a 5-*μ*m filter at 2–4 mL/min over 30 minutes per manufacturer's instructions. Thirty minutes after completion of SPIO infusion, subjects were scanned supine in a superconducting MR whole body system at 3T (GE Signa EXCITE HD, GE Medical Systems, Milwaukee, WI), with an 8-channel torso phase-array coil and a dielectric pad centered over the liver. Gadolinium-DTPA (gadopentetate dimeglumine, Magnevist, Bayer HealthCare Pharmaceuticals, Wayne, NJ) was injected intravenously (0.1 mmol/kg). Using a 2D chemically fat-saturated fast spoiled gradient-recalled echo (FSPGR) sequence without parallel imaging, four sets of axial CCE images of the liver were acquired during separate 18–28 second breath-holds, 4–10 minutes after Gd injection. In this 6-minute window, enhancement of the liver by the two agents (SPIO and Gd) is subjectively constant according to our clinical experience of CCE MR imaging in cirrhotic and non-cirrhotic livers; moreover, the T1- and T2^*∗*^ shortening effects of gadopentetate and ferumoxides in liver may be assumed stable over this period from the known liver clearance rates of these agents [31–33]. The four image sets were acquired to help ensure that at least one set was free of visible motion artifacts. Imaging parameters included TR 100 ms, TE 6 ms, FA 70°, slice thickness 4 mm, interslice gap 4 mm, number of slices 5, and bandwidth 130 Hz/pixel. Two of the four image sets were acquired with 384 × 224 and two with 384 × 256 matrix. Field-of-view was adjusted to accommodate body habitus and breath-hold capacity. These parameters were selected to provide simultaneous T1- and T2^*∗*^-weighting to exploit Gd- and SPIO-enhancement, respectively; adequate signal-to-noise ratio; high spatial resolution; and relatively short acquisition time. The Food and Drug Administration (IND number 75,579) approved off-label use of Magnevist-Feridex combined contrast for this research study.

### 2.5. Image Processing and Texture Analysis

A radiology resident (TY) and a trained research assistant (CC) analyzed the CCE images without knowledge of clinical or biopsy findings. From the four CCE image sets, the set with the highest resolution and subjectively least motion artifact was selected. Representative CCE images of the liver (1–5 sections per subject) were exported in DICOM format. Using MATLAB (Mathworks, Natick, MA), a total of five nonoverlapping rectangular regions-of-interest (ROIs) of size >100 mm^2^ were placed per subject within areas of subjectively uniform texture in the right hepatic lobe (Couinaud segments IV–VIII), avoiding artifacts, bile ducts, and vessels. Each ROI image was standardized by rotating to the Cartesian coordinate system with zero tilt-angle, interpolating to 0.5 mm/pixel resolution, removing bilinear spatial trend of signal intensities, and scaling to 0-1 intensity range.

Gradient and Laplacian transformations (1st and 2nd spatial derivatives) were applied to each standardized ROI to generate additional “edge-enhanced” and “zero-crossing” texture patterns. For each untransformed (original) and transformed (gradient, Laplacian) ROI, 55 texture features were calculated as detailed in the supplementary materials available online at  http://dx.doi.org/10.1155/2014/387653. These texture features represented five texture feature classes: pixel intensity histogram, Gaussian mixture model, autocorrelation, cooccurrence matrices, and Voronoi polygons. These classes were selected based on the expected imaging characteristics of fibrosis texture, as explained in the supplementary materials. For each subject, the texture features were averaged across the five ROI's to generate a set of 165 average texture features.

### 2.6. Statistical Analyses

#### 2.6.1. Comparison of Histologic Scores

For each subject, the average, standard deviation (STD), and range of the Metavir scores of the three pathology readers were calculated. The interreader agreement was assessed by intraclass correlation coefficient (ICC, two-way analysis for precise agreement) and their 95% confidence intervals (CIs) were calculated. ICC was also calculated for each pair of readers. The average Metavir scores of the three readers were compared to %-collagen scores using Pearson correlation analysis.

#### 2.6.2. Comparison of Texture and Histology

A biostatistician (TW) performed statistical analysis using the 165 texture features to predict qualitative (Metavir) and quantitative (%-collagen) fibrosis scores. A path-following algorithm for *L*_1_ regularized linear model called GLM-path [34] with a Gaussian link (i.e., linear regression) was used to identify the optimal linear model of texture features that minimized the fibrosis prediction error for each number of predictors (i.e., features). The optimal number of predictors was determined by Akaike Information Criterion (AIC) [35]. Using the qualitative and quantitative fibrosis scores as the reference, two texture-based fibrosis prediction models were constructed, respectively. For each subject, the predicted qualitative (Metavir) and quantitative (%-collagen) fibrosis scores were calculated using respective prediction models.

Pearson's correlation was used to evaluate the strength of the relationship between the predicted and histologic scores. Additionally, the performance of each prediction model for dichotomized classification was assessed using receiver-operating-characteristics (ROC) analysis using the average histologically determined Metavir score as the reference standard. At each of four classification thresholds (Metavir F1, F2, F3, and F4 for qualitative scoring; 5, 10, 15, and 20% collagen for quantitative scoring), the classification accuracy, sensitivity, and specificity (and their CIs) were calculated at the predicted fibrosis score cutoff value that maximized the sum of sensitivity and specificity.

The regularization employed by the GLM-path algorithm is designed to minimize prediction error over independent validation datasets [34]. Therefore no dedicated validation procedure was performed in this proof-of-concept study. However, the algorithm may not necessarily minimize the prediction error of the test dataset itself; thus, some degree of mismatch between the predicted and actual fibrosis scores is expected.

## 3. Results

### 3.1. Subjects

Between August 2007 and March 2009, 52 newly diagnosed HCV-positive adults (age 51.2 ± 6.3 years, 38 male, 12 female) with recent or planned liver biopsy were recruited for CCE imaging. Six subjects were excluded (Table 1). The remaining 46 subjects formed the study group. All subjects completed the MR examination without serious adverse effects. At least one CCE image set was subjectively adequate in quality for further image analyses in each subject.

### 3.2. Qualitative versus Quantitative Histology

**Figure 3:**
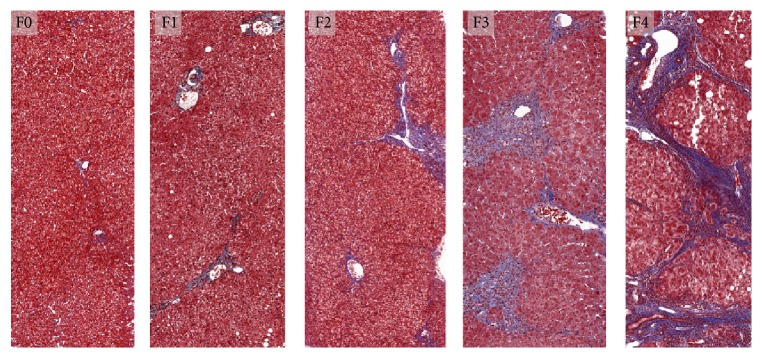
Histologic assessment of liver HCV-related fibrosis. Liver biopsy specimen from subjects with chronic HCV infection, stained with Masson-trichrome. F0 (absent fibrosis), F1 (stellate enlargement of portal tracts), F2 (enlarged portal tracts with rare septa), F3 (numerous septa without cirrhosis), and F4 (cirrhosis) according to Metavir scoring system. Trichrome stains fibrosis blue.

Examples of biopsy specimens are shown in Figure 3. The histology specimen's average ± STD [range] of the total length and the number of portal triads were 21.9 ± 9.8 mm [6.7–44.2] and 14.2 ± 6.0 [4–28], respectively.

**Figure 4:**
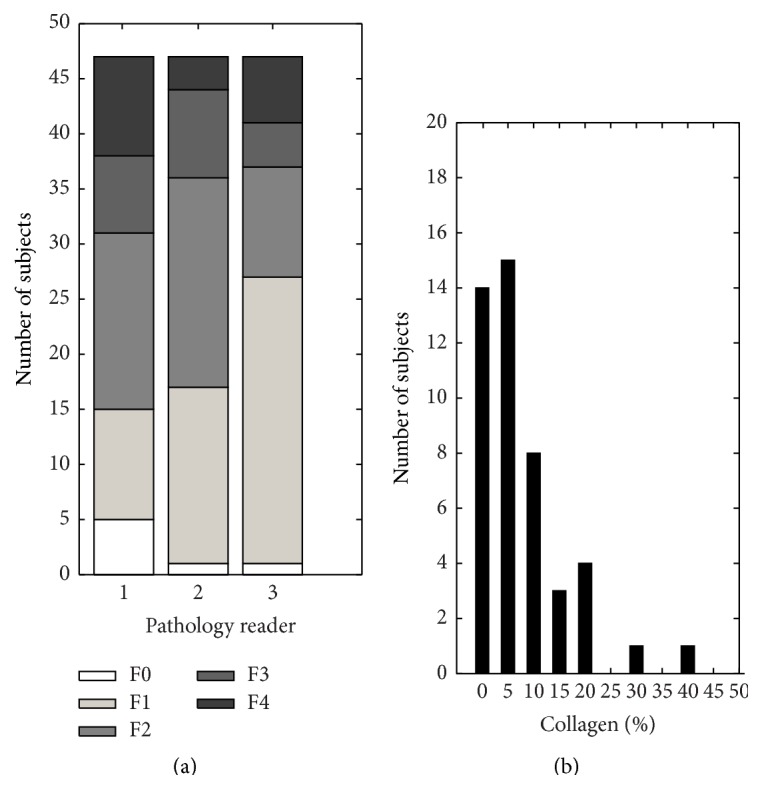
Fibrosis severity distribution. The study group's histograms by Metavir fibrosis score (left) and %-collagen (right). The most common Metavir fibrosis score was F1 or F2 depending on the reader. Nine subjects (19%; 9/46) had a score of F4 (cirrhosis) from at least one reader. %-collagen is rounded to the nearest 5%.

Figure 4(a) shows the histogram of qualitative Metavir scores assigned by the three readers. The 3-reader agreement was good with ICC of 0.772 (95% CI [0.653–0.859]). Pairwise ICCs were 0.727, 0.768, and 0.831, depending on the reader pairs. All readers agreed that all biopsy specimens were adequate.

**Figure 5:**
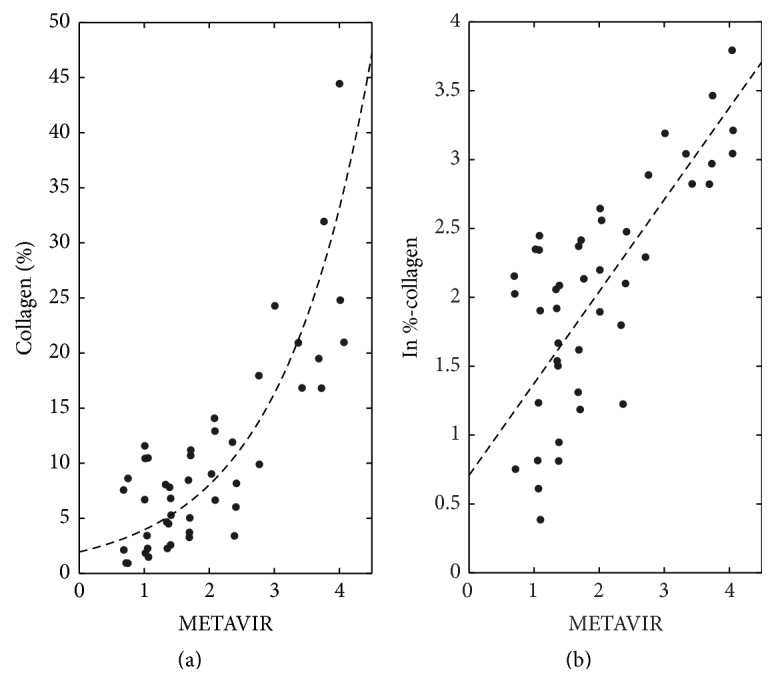
Comparison between quantitative versus qualitative histology. Average Metavir of 3 pathology readers versus (a) raw %-collagen (b) and natural logarithm of %-collagen. As shown in (a), the relationship between quantitative and qualitative histology scores is curvilinear. Pearson's correlation coefficient (*r*) of plot B is 0.81, with *P* < 0.001.

Figure 4(b) shows the histogram of quantitative %-collagen rounded to the nearest 5%. Over half the subjects had rounded %-collagen ≤5%. As shown in Figure 5 the relationship between the qualitative (average Metavir) and quantitative (%-collagen) scores was curvilinear, as has been observed by others [36, 37]. Log-linear plot of quantitative (*y*-axis) and qualitative (*x*-axis) scores demonstrated significant linear correlation with Pearson's *r* = 0.81 (*P* < 0.001).

### 3.3. Image Texture versus Histology

**Figure 6:**
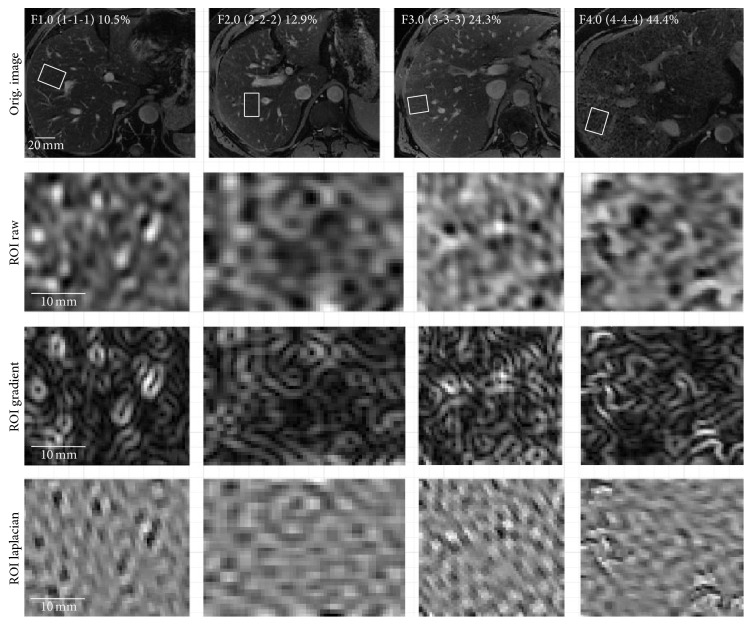
Regions-of-interest on CCE MR images in four subjects. Regions-of-interest drawn on CCE MR images in four representative subjects. Average Metavir (and individual reader METAVIR) and %-collagen scores are shown. Subjectively, high-signal reticular texture becomes increasingly conspicuous and disorganized with increasing Metavir and %-collagen scores. CCE: combined contrast enhanced.

The liver image textures of representative subjects are shown in Figure 6 with their respective qualitative (Metavir) and quantitative (%-collagen) scores.

**Figure 7:**
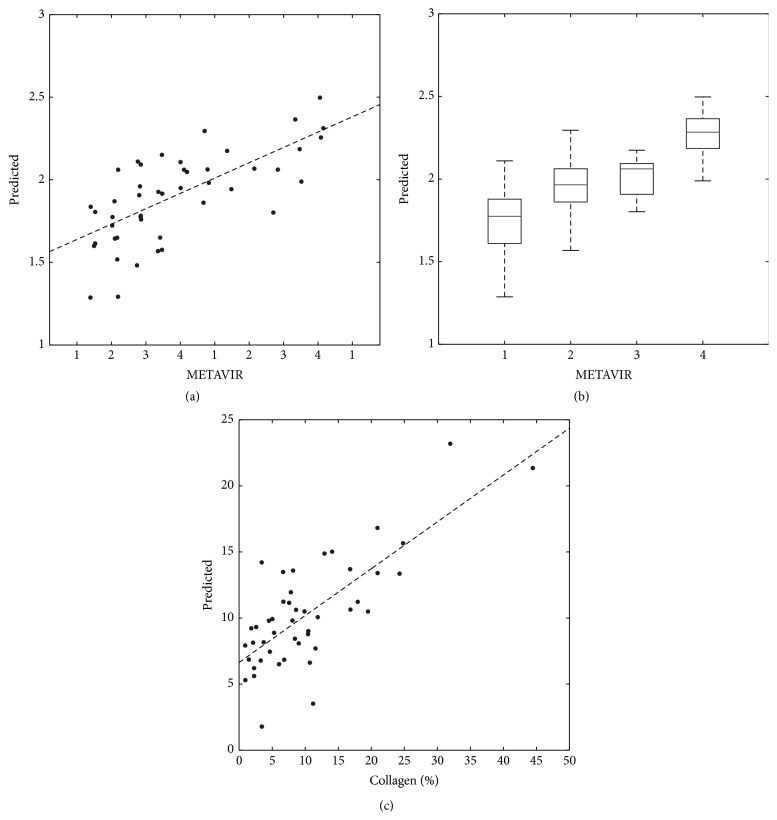
Texture versus histologic fibrosis scores. (a) The correlation between the texture and qualitative histology score (average Metavir) is statistically significant with 0.698 (*P* < 0.001) with best-fit line of slope 1.546 and intercept 0.186. (b) Box-plot of texture score versus rounded average Metavir. Spearman's correlation is significant at *ρ* = 0.635 (*P* < 0.001). (c) The correlation between texture and quantitative histology score (percent-fibrosis) is statistically significant with 0.767 (*P* < 0.001), with best-fit line of slope 0.355 and intercept 6.636. The nonunit slope and nonzero intercept are attributable in part to the regularization procedure employed by the GLM-path algorithm (see text).

Using qualitative histology as the reference, GLM-path analysis identified a set of 6 texture features predictive of Metavir fibrosis scores (Table 2). As shown in Figure 7(a), the Metavir score predicted by a 6-feature model linearly correlated with the average Metavir scores of the three readers with *r* = 0.698  (*P* < 0.001).  Table 3 summarizes the ROC analysis results at each classification threshold. AUCs were 0.814–0.976, sensitivities 0.659–1.000, specificities 0.778–0.930, and accuracies 0.674–0.935, depending on the classification threshold.

Using quantitative histology as the reference, GLM-path analysis identified another set of 6 texture features predictive of %-collagen scores (Table 4). As shown in Figure 5 (LEFT) the %-collagen score predicted by the 6-feature model linearly correlated with %-collagen score of histology with *r* = 0.757  (*P* < 0.001).  Table 5 summarizes the ROC analysis results at threshold values at 5, 20, 15, and 20% fibrosis. AUCs were 0.742–0.950, sensitivities 0.688–1.000, specificities 0.679–0.857, and accuracies 0.696–0.848, depending on the classification threshold.

**Figure 8:**
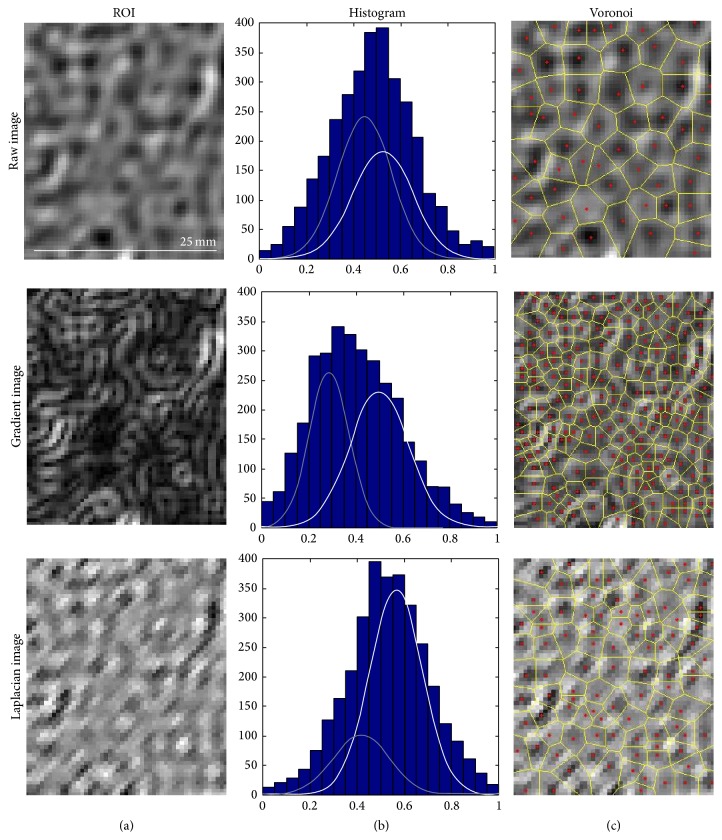
Illustrative examples of texture feature classes for fibrosis prediction. A 54 year-old female with cirrhosis. (c) Standardized regions of interest (ROI) images, without transformation (raw), with gradient and Laplacian transformations. (b) Corresponding pixel intensity histogram and its Gaussian mixture model fit with two normal populations. (a) Voronoi polygons constructed on the corresponding ROI images. These texture classes were found to be predictive of liver fibrosis on CCE images (see text). For each texture class (intensity histogram, Gaussian mixture, and Voronoi polygons), relevant statistics were calculated as detailed in supplementary materials and were used for fibrosis prediction.

Identified texture features were similar but not identical between qualitative and quantitative prediction models (Tables 2 and 4). Two classes of texture features were common to both Gaussian-mixture model and Voronoi polygons. One class of texture features (pixel intensity histogram) was predictive only for qualitative scores. Texture features of both untransformed and transformed ROI images were found to be predictive. For illustration purposes, these texture classes derived from a ROI in a cirrhotic subject are shown in Figure 8.

## 4. Discussion

This study prospectively assessed liver fibrosis in HCV-infected adults noninvasively using quantitative texture analysis of CCE MR images. Liver biopsy was used as the reference standard. Fibrosis severity was scored qualitatively (Metavir) and quantitatively (%-collagen). The study design closely simulated a typical clinical situation, in which a newly diagnosed HCV-positive patient without clinically overt cirrhosis requires assessment of liver fibrosis.

We utilized a CCE MR imaging technique, in which SPIOs and an extracellular Gd-based agent are administered sequentially. Prior studies suggested complimentary effects of SPIO and Gd for visualizing fibrosis. SPIOs accumulate by phagocytosis in Kupffer cells in the hepatic lobules, causing T2^*∗*^-related negative enhancement. Extracellular Gd-based agents such as Gd-DTPA distribute to the interstitial space of the fibrotic perilobular septa, causing T1-related positive enhancement. The result is a linear meshwork of high-signal perilobular septa against a background of low-signal lobules, producing a reticular texture pattern that subjectively becomes more conspicuous with increasing fibrosis severity [26, 27].

We found that CCE MR image texture of the liver can be objectively quantified to predict fibrosis severity. The abnormal texture was detectable at early fibrosis stage, for example, F > 2 Metavir score or >15%-collagen with accuracy of 0.826 and 0.783, respectively. The predicted fibrosis scores correlated with but did not exactly match the corresponding histologically determined scores. The imperfect agreement between predicted and actual fibrosis scores is likely due to three factors: intrinsic inaccuracy of the texture-based technique used in our study, expected mismatch due to the regularization procedure employed by GLM-path (explained earlier), and intrinsic inaccuracy of liver biopsy as a reference standard (explained later).

Gaussian mixture models and Voronoi polygons were found to be predictive texture classes by both qualitative and quantitative histology prediction models. This is consistent with the postulated complimentary effects of SPIO and Gd producing the reticular enhancement pattern in fibrotic livers. A Gaussian mixture model fits two normal distributions, each with its own mean and variance, to the overall pixel intensity histogram. On a CCE image, the liver contains two populations of pixels, one comprised of low-signal SPIO-containing pixels devoid of fibrosis and the other of high-signal Gd-containing pixels in fibrotic septa. With progression of fibrosis, the proportion of high-signal Gd-containing pixels (i.e., fibrosis) increases and, therefore, the pixel intensity histogram is better fitted by a mixture of two Gaussian distributions than a single Gaussian. The Voronoi polygon algorithm generates a tessellation of polygons that “carves” the liver parenchyma into low-intensity nodules, thereby objectively modeling the reticular texture seen subjectively in progressive fibrosis.

Another MR-based technique, MR elastography (MRE), is increasing in popularity and availability for noninvasive assessment of liver fibrosis. This technique measures the biomechanical stiffness of the liver, which increases as a consequence of fibrosis [38]. In a retrospective study in HCV-infected population [39], the reported AUC, sensitivity, and specificity of MRE in detecting clinically significant fibrosis (F ≥ 2) was 0.986, 0.885, and 1.00, respectively, similar to slightly higher than those of the CCE texture method. However, texture-based methods may have a theoretical advantage of more direct visualization of fibrosis while MRE measures the tissue biomechanical sequela of fibrosis. Another practical advantage of texture-based methods is that they can be implemented on any clinical scanner using standard sequences, while MRE requires dedicated hardware (mechanical wave transducer) and sequences. Disadvantages of texture-based methods are the need for intravenous access and injection of contrast agents, including two agents for the CCE technique described here. Also, visualization of subtle reticulations associated with early fibrosis is sensitive to patient motion; consequently we obtained four CCE image sets in separate breath-holds, to ensure that at least one set was motion-free. As motion correction/minimization techniques become more robust and clinically available, it may be possible to acquire images during free breathing with higher signal-to-noise ratio and spatial resolution.

A limitation of this study is the use of single liver biopsy as the reference standard. A typical core biopsy (~30 mm^3^) samples only 1/50,000 of the liver and is significantly smaller than the imaging ROI (>400 mm^3^) used in the texture analysis. Also, biopsied sites are difficult to colocalize with imaging ROIs, which is relevant because the severity of fibrosis can be heterogeneous across the liver. An error frequency of up to 33% has been reported for differences in ≥ one fibrosis stage and cirrhosis may be missed in 10–30% of blind biopsies [40]. Thus even a “perfect” fibrosis prediction method may have only moderate observed accuracy in binary classification if single-biopsy histology is used as the reference. Obtaining multiple biopsies may reduce errors in the reference standard, but increases the complication risk and was not feasible in this study. Considering these limitations, moderate accuracy of CCE MR imaging is appropriate and expected. Determination of the true accuracy of fibrosis imaging may require histologic evaluation of larger specimens than those obtained by percutaneous biopsy. Another consideration for the accuracy of biopsy as the reference standard is observer bias [10]. To minimize the observer bias and increase the accuracy of fibrosis staging, this study used the average Metavir score of three hepatopathologists' independent interpretations as the reference standard. While averaging of an ordinal fibrosis score is less than ideal, it is arguably the most valid fibrosis severity metric available from a single biopsy specimen. Such a practice is not uncommon in hepatology literature [41–44]. As average Metavir score is expected to preserve the rank-order relationship of the fibrosis severity, it should be sufficient to mathematically construct a valid fibrosis prediction model.

While this study suggested a promising indication for SPIO agents in liver fibrosis imaging, ferumoxides were withdrawn from the US market in 2009. Recently, another intravenously injectable SPIO-based drug, ferumoxytol (Feraheme, AMAG Pharmaceuticals, Lexington, MA) has been FDA-approved for iron-deficiency therapy and early data on its application as contrast agent for MR imaging are promising [45]. While this new drug likely has similar negative-contrast effects in the liver as ferumoxides, further studies will be necessary to evaluate its effectiveness in CCE imaging.

In summary, this proof-of-concept prospective study showed that CCE MR imaging and quantitative texture analysis may permit noninvasive assessment of liver fibrosis in HCV-infected adults. MR image texture is a potential noninvasive biomarker of liver fibrosis and, with further technical refinement and validation, may provide a new tool in clinical management and research in HCV-infected patients.

## Supplementary Material

For interested readers, the technical details of the texture analyses, including image normalization, transformation, texture feature calculations, and relevant references, are included in the supplemental material.

## Figures and Tables

**Table 1:** Selection criteria.

Inclusion criteria	Exclusion criteria
(i) Age >18 years (ii) Newly diagnosed HCV infection, without clinically overt cirrhosis (iii) Recent or planned biopsy^1^ (iv) Willing and able to undergo CCE MRI exam within 30 days of biopsy (v) Willing and able to undergo phlebotomy for estimated GFR determination within 30 days of biopsy	(i) Estimated GFR <60 mL/mL (*N* = 0 potential subjects) (ii) Imaging not performed within 30 days of biopsy (*N* = 2) (iii) Nondiagnostic biopsy or trichrome slide unavailable (*N* = 2) (iv) Contraindication to MR exam (*N* = 1)^2^ (v) Lack of intravenous access (*N* = 1) (vi) History of severe allergic reaction or anaphylaxis (*N* = 0) (vii) History of liver diseases other than HCV including iron overload (*N* = 0) (viii) Severe claustrophobia (*N* = 0) (ix) Pregnant or nursing mother (*N* = 0)

^1^Biopsies were performed for clinical care. ^2^Due to intraorbital shrapnel. GFR: glomerular filtration rate. Parenthesis () contains the number of potential subjects excluded for the criterion.

**Table 2:** Fibrosis prediction model parameters (texture versus Metavir).

	Source image	Texture class	Texture feature
1	Original	Pixel intensity histogram	Mean pixel intensity
2	Original	Gaussian mixture model	STD of the lower intensity pixels
3	Original	Gaussian mixture model	AIC of two-Gaussian fit/AIC of single-Gaussian fit
4	Original	Voronoi polygons	STD of the 1st order inertial moment
5	Gradient	Voronoi polygons	Mean of the 2nd order inertial moment
6	Laplacian	Pixel intensity histogram	Mode/interquartile range

Six most predictive texture features, from strongest to weakest. Keys: STD: standard deviation, AIC: Akaike Information Criterion, inertial moments: mathematical description the shape/area of the Voronoi polygons (see supplementary materials).

**Table 3:** Receiver operating characteristics (texture versus Metavir).

Classification	Cutoff	Area under curve	Sensitivity	Specificity	Accuracy
F <1 versus F ≥1	1.805	0.814 [0.654 0.975]	0.659 [0.513 0.804]	0.800 [0.449 1.000]	0.674 [0.524 0.797]
F <2 versus F ≥2	1.916	0.889 [0.783 0.994]	0.895 [0.757 1.000]	0.778 [0.621 0.919]	0.826 [0.686 0.916]
F <3 versus F ≥3	2.060	0.862 [0.701 1.000]	0.778 [0.506 1.000]	0.784 [0.651 0.916]	0.783 [0.615 0.867]
F <4 versus F = 4	2.174	0.976 [0.855 1.000]	1.000 [0.907 1.000]	0.930 [0.854 1.000]	0.935 [0.788 0.974]

Cutoff: the operating point on the ROC curve closest to (0,1), the point of maximum sensitivity and specificity. [  ]—95% confidence interval. The mismatch between the texture-based cutoff and the histologic classification threshold is expected (see text).

**Table 4:** Fibrosis prediction model parameters (texture versus %-collagen).

	Source image	Texture class	Texture feature
1	Original	Gaussian mixture model	STD of the lower intensity pixels
2	Original	Voronoi polygons	Mean of the 2nd order inertial moment
3	Original	Voronoi polygons	STD of the 1st order inertial moment
4	Gradient	Voronoi polygons	Mean of the 2nd order inertial moment
5	Gradient	Gaussian mixture model	STD of the lower intensity pixels
6	Laplacian	Voronoi polygons	Mean of the 3rd order inertial moment

Six most predictive texture features, from strongest to weakest. Keys: STD: standard deviation, AIC: Akaike Information Criterion, inertial moments: mathematical description the shape/area of the Voronoi polygons (see supplementary materials).

**Table 5:** Receiver operating characteristics (texture versus %-collagen).

Classification	Cutoff	Area under curve	Sensitivity	Specificity	Accuracy
<5% versus ≥5%	9.315	0.806 [0.680 0.932]	0.688 [0.527 0.848]	0.857 [0.674 1.000]	0.739 [0.592 0.850]
<10% versus ≥10%	9.807	0.742 [0.589 0.895]	0.722 [0.515 0.929]	0.679 [0.506 0.852]	0.696 [0.547 0.815]
<15% versus ≥15%	10.500	0.894 [0.758 1.000]	0.900 [0.714 1.000]	0.750 [0.609 0.891]	0.783 [0.638 0.884]
<20% versus ≥20%	11.234	0.950 [0.826 1.000]	1.000 [0.907 1.000]	0.825 [0.707 0.943]	0.848 [0.686 0.916]

Cutoff: the operating point on the ROC curve closest to (0,1), the point of maximum sensitivity and specificity. [  ]—95% confidence interval. The mismatch between the texture-based cutoff and the histologic classification threshold is expected (see text).
