# Supplementary material for: Evaluation of Liver Fibrosis Using Texture Analysis on Combined-Contrast-Enhanced Magnetic Resonance Images at 3.0T
Source: Biomed Res Int. 2015 Sep 1;2015:387653. doi: 10.1155/2015/387653 (PMC4569760; doi:10.1155/2015/387653)
Supplement: Supplementary file 1 — For interested readers, the technical details of the texture analyses, including image normalization, transformation, texture feature calculations, and relevant references, are included in the supplemental material. [file 387653.f1.pdf]

APPENDIX

Introduction

Image texture, defined as a "function of the spatial variation in pixel intensities" (1), is used in this study to detect the presence of liver fibrosis and predict its severity. On combined contrast enhanced (CCE) magnetic resonance (MR) images of the liver, image texture within regions of interest (ROIs) were objectively quantified using texture analysis. The five texture classes considered in this study were statistics of the pixel intensity histogram, Gaussian-mixture model (2), autocorrelation (3), gray-level co-occurrence matrices (4), and Voronoi polygons (5). This appendix discusses technical and computational details.

The choice of these texture classes and their calculation methods was made in consideration for the expected mathematical and morphological properties of the liver fibrosis texture. Desired texture features for CCE images would characterize high intensity reticulations, low intensity nodules, and the contrast between these two areas. The texture features would be locally uniform, invariant under linear transformations of the intensity value, and invariant under certain spatial transformations of the ROI (e.g. translation, reflection, rotation). Based on these expectations, the following calculations were performed.

Texture Analysis

Image Normalization

Each source ROI image was normalized as follows. First, the rectangular ROI was rotated such that the rectangle's edges were parallel to Cartesian coordinate axes. Then, the rotated images were re-gridded and interpolated at 0.5mm pixel resolution. To correct for spatial drift in pixel intensity within the ROI, bi-linear trend was estimated and removed using 2D linear regression. The pixel intensity scale was corrected to the standardized range [0 1]. This normalization was necessary to ensure that the calculated texture features are comparable across different ROIs, acquisitions, and subjects.

Image Transformations

Each normalized, source ROI image was transformed as follows. Let F(i,j), the pixel intensity at i-th column and j-th row, be the standardized source ROI image. The gradient image G(i,j) was calculated as the magnitude of the gradient vector at each pixel location, $G=|\nabla F|$, where ∇ denotes the gradient operator. Similarly, the Laplacian image L(i,j) was calculated as $L=\nabla^2 F$, where ∇^2 is the "del" operator. The gradient and Laplacian images are also referred to "edge-enhanced" and "zero-crossing" images of the original ROI images. These have visually distinct (but related) patterns from the original image (Error! Reference source not found.) and thus may be helpful in characterize fibrosis textures.

Pixel Intensity Histogram

The pixel intensity histogram of the ROI image (F, G, or L) was characterized by the following parameters: mean, standard deviation, coefficient of variation, kurtosis, skewness, inter-quartile range (IQR), median/IQR, mode/IQR, and entropy, a measure of "randomness" (6). These represent the texture features 1 through 9 (T1-9).

Gaussian-Mixture Models

The intensity histogram was fitted to two distribution models, (1) single Gaussian and (2) a mixture of two Gaussians. The two-Gaussian model considers a histogram h(x) as a sum of two normal populations (lower and higher intensity populations), each with its own mean and variance

$$h(x) = p_L\, g_L(x;\, \mu_L,\sigma_L) + p_H\, g_H(x;\, \mu_H,\sigma_H)$$

where h(x) is the overall probability density function for intensity value x, g is the normal density function with mean μ and standard deviation (std) σ. The mixing proportion p indicates the relative abundance of the lower and higher intensity populations. For each histogram, the model parameters (p_L, μ_L, σ_L and p_H, μ_H, σ_H) were estimated (T10-15), and the Mahalanobis distance (T16) between g_L and g_H, as well as Akaike information criterion (AIC) of the fit, were calculated using the Gaussian-Mixture Model (GMM) package in MATLAB statistical toolbox. The AIC for the single-Gaussian model fit was also calculated, and the goodness-of-fit of the two- vs. one-Gaussian models was compared by the ratio AIC_2/AIC_1 (T17).

Autocorrelation

The 2-dimensional autocorrelation function of the ROI image was calculated within ±10mm spatial offsets in x- and y-directions as:

$$R(i',j') = \frac{1}{N}\sum_{i}\sum_{j} F(i,j)F(i-i',j-j')/F(i,j)^{2}$$

where N is the number of pixels in the ROI, and i' and j' are the offsets in the x- and y-directions. This range of offsets [-10mm, +10mm] was chosen under the assumption that the spatial structure of fibrosis has scales <10mm. On the calculated autocorrelation map, the iso-contour loci of value 1/e were fitted to an ellipse. The long- and short-axes length (T18, 19), average axes length (T20), and the long-short axis ratio (T21) were calculated to assess the spatial scale and orientation of the autocorrelation.

Co-occurrence Matrix

Gray-level co-occurrence matrices (GLCMs) were generated within ±4 pixel offsets (±2mm) in x- and y-direction. For each of the 16 offsets, the contrast, correlation, energy, homogeneity, and entropy of the corresponding GLCM were calculated using the MATLAB image processing toolbox. To preserve rotational invariance, these GCLM features were averaged according to the offset pixel distances (0-2], (2-4], (4-6], (6-8], where the brackets ( ] define an interval open on the left and closed on the right. This procedure generated 20 (5 features x 4 offset distances) texture features (T21-41).

Voronoi Polygons

The vertices of Voronoi polygons were chosen at 2D local intensity minima of the ROI image. Using these vertices as seeds, a tessellation of Voronoi polygons were generated to fill the ROI using a MATLAB Voronoi algorithm. The average and the STD of number of edges (T42, 43), length of edges (T44, 45), seed density (T46, 47), and edge density (T48-49) were computed. The polygon's 0th to 3rd-degree inertial moments (T50-55) were also computed using a previously described algorithm (7).

Summary

For each ROI location, three ROI images were generated (F, G, and L). For each ROI image, 55 texture features were computed as detailed above. Thus, a total of 165 texture features were computed per ROI location. These texture features were averaged across five non-overlapping ROI locations within the subject's liver. The average 165 texture features of the individual subject served as the input variables for the multiple regression model described in the main text.

References

1. Tuceryan M, Jain AK. Texture Analysis. In: Chen CH, Pau LF, Wang PSP, eds. The Handbook of Patten Recognition and Computer Vision. 2nd ed: World Scientific Publishing Co., 1998; pp 207-248.
2. McLachlan G, Peel D. Finite Mixture Models: John WIley & Sons, Inc., 2000.
3. Lin H, LL W, SN Y. Extracting periodicity of a regular texture based on autocorrelation functions. Pattern Recognition Letters 1997; 18:433-443.
4. Haralick R, Shanmugam K, Dinstein I. Textural Features for Image Classification. IEEE Transactions on Systems, Man and Cybernetics 1973; 3:610-620.
5. Voronoi G. Nouvelles applications des paramètres continus à la théorie des formes quadratiques. Journal für die Reine und Angewandte Mathematik 1907:97-178.
6. Shannon CE. A Mathematical Theory of Communication. Bell System Technical Journal 1948; 27:pp 379-423, 623-656.
7. WIlson Jr. HB, Farrior DS. Computation of geometrical and inertial properties for general areas and volumes of revolution. Computer Aided Design 1976; 8:257-263.
